# Senolytics attenuates influenza- induced muscular decline in aged mice

**DOI:** 10.1093/geroni/igaf122.3652

**Published:** 2025-12-31

**Authors:** Nagaraju Marka, Andreia Cadar, Zena Haddad, Anchala Anil Rao, Rakshit Raj Bisoi, Erica Lorenzo, Laura Haynes, Jenna Bartley

**Affiliations:** University of Connecticut Health, University of Connecticut Health, Farmington, Connecticut, United States; University of Connecticut Health, University of Connecticut Health, Farmington, Connecticut, United States; University of Connecticut Health, University of Connecticut Health, Farmington, Connecticut, United States; University of Connecticut Health, University of Connecticut Health, Farmington, Connecticut, United States; University of Connecticut Health, University of Connecticut Health, Farmington, Connecticut, United States; University of Connecticut Health, University of Connecticut Health, Farmington, Connecticut, United States; University of Connecticut Health, University of Connecticut Health, Farmington, Connecticut, United States; University of Connecticut School of Medicine, University of Connecticut Health, Connecticut, United States

## Abstract

Influenza (flu) remains a major burden for older populations with increased morbidity and mortality. In older people, flu infection can lead to loss of physical function. In preclinical murine studies, the Bartley lab previously determined that flu leads to functional decline, muscle-localized inflammation, and muscle degradation and atrophy; and that these effects are exaggerated and prolonged with aging. Cellular senescence accumulate with aging and leads to excessive inflammation via the senescent-associated secretory phenotype (SASP). Treatment with senolytics, dasatinib and quercetin (D+Q), eliminates senescent cells and improves the health span, metabolic functions, reduces muscle inflammation and promotes satellite cell proliferation in aging animal models. Hence, we aimed to determine if pretreatment with D+Q could protect against flu-induced muscle declines in aging. Aged (18-20M) C57BL6/J male and female mice were treated with D+Q prior to sublethal flu infection. Immune responses, physical function, and skeletal muscle parameters were assessed prior to and following flu challenge. D+Q did not impact flu-induced weight loss, lung viral load, and flu-specific CD4 and CD8 T cells responses in the mediastinal lymph node and spleen. However, D+Q treatment mitigated flu-induced functional declines in both male and female with increased grip strength compared to vehicle control. In male D+Q treatment led to reduced gene expression of muscle atrophy markers Atrogin-1 and MuRF-1 compared to vehicle treated mice at 11- days post-infection. In totality, our results suggest senolytics may protect against flu-induced muscular declines without impacting immune responses in aged mice, which may offer insights for potential therapeutic implications.

